# Plates Illustrative of Wilson on Diseases of the Skin

**Published:** 1858-05

**Authors:** 


ART. V.—*Plates Illustrative of Wilson on Diseases of the Skin.* Fourth edition. Philadelphia: Blanchard & Lea. 8vo. 1857.

IN the September number of this journal we introduced to our readers the fourth edition of Mr. Wilson's work on the Diseases of the Skin, and we have just received from the distinguished publishers a copy of plates illustrative of the various affections therein described. These plates, of octavo size, are nineteen in number, and comprise numerous figures, most of them colored in the highest style of the art. In the present edition are included Mr. Wilson's drawings of constitutional syphilis and syphilitic eruptions, thus rendering the work more complete and valuable. The letter-press, although concise, is sufficiently clear and expressive of the objects which it is intended to fulfil. Knowing how little attention is paid to this class of affections by the physicians of this country, we can safely recommend this work to their consideration as a valuable clinical guide.

